# Continuous Reduction of Protein-Bound Uraemic Toxins with Improved Oxidative Stress by Using the Oral Charcoal Adsorbent AST-120 in Haemodialysis Patients

**DOI:** 10.1038/srep14381

**Published:** 2015-09-23

**Authors:** Suguru Yamamoto, Junichiro J. Kazama, Kentaro Omori, Koji Matsuo, Yoshimitsu Takahashi, Kazuko Kawamura, Takayuki Matsuto, Hiroshi Watanabe, Toru Maruyama, Ichiei Narita

**Affiliations:** 1 Division of Clinical Nephrology and Rheumatology, Niigata University Graduate School of Medical and Dental Sciences, Niigata 951-8510, Japan; 2 Division of Blood Purification Therapy, Niigata University Medical and Dental Hospital, Niigata 951-8520, Japan; 3 Omori Clinic, Niigata 950-0909, Japan; 4 Division of Clinical Preventive Medicine, Niigata University Graduate School of Medical and Dental Sciences, Niigata 951-8510, Japan; 5 Department of Biopharmaceutics, Graduate School of Pharmaceutical Sciences, Kumamoto University, Kumamoto 862-0973, Japan

## Abstract

Accumulation of protein-bound uraemic toxins (PBUTs) is one of the reasons for the development of uraemia-related complications including cardiovascular disease; however, conventional haemodialysis is limited in its ability to remove PBUTs. We aimed to examine whether the oral charcoal adsorbent AST-120 has an additive effect on PBUT removal in haemodialysis patients. During the 4-week study, anuric patients undergoing haemodialysis received AST-120 (6 g/day) in the last 2 weeks (n = 10) or the first 2 weeks (n = 10). Serum levels of total and free PBUTs such as indoxyl sulfate, *p-*cresyl sulfate, and phenyl sulfate at the pre- and postdialysis sessions were measured before and after AST-120 use and after discontinuation. Levels of the oxidative stress markers oxidized albumin and 8-isoprostane were also measured. AST-120 use induced dramatic reduction of indoxyl sulfate (total, 45.7% [33.2–50.5%]; free, 70.4% [44.8–79.8%]), *p-*cresyl sulfate (total, 31.1% [25.0–48.0%]; free, 63.5% [49.3–70.9%]), and phenyl sulfate (free, 50.6% [32.3–71.2%]) levels; however, this effect disappeared after the discontinuation of AST-120. AST-120 use also induced substantial reduction of the oxidized albumin and 8-isoprostane levels. In conclusion, oral administration of AST-120 had additive effects on the continuous reduction of some PBUTs in anuric patients undergoing haemodialysis.

An accumulation of uraemic toxins is associated with chronic kidney disease (CKD)-related complications including cardiovascular disease, especially in patients undergoing dialysis treatment^1^,^2^,^3^. With the development in dialysis therapies, removal of low- and middle molecular-weight water-soluble molecules has been improving; however, conventional haemodialysis treatment is limited in its ability to remove protein-bound uraemic toxins (PBUTs) owing to binding to large molecular proteins^4^. For example, the protein-binding rate of indoxyl sulfate (IS), a representative PBUT, is 97.7%, and the reduction rate with regular haemodialysis treatment is 31.8%^5^. Several clinical studies suggested that IS and *p*-cresyl sulfate (PCS) were related to cardiovascular mortality in CKD and dialysis patients^1^,^2^, and the accumulation of IS or PCS is related to the increase in oxidative stress^3^,^6^,^7^. Thus, additional therapeutic strategies will be needed to reduce PBUT levels for preventing CKD-related complications.

The oral charcoal adsorbent AST-120 is known to reduce the serum levels of IS through the adsorption of indole converted from dietary tryptophan in the gastrointestinal tract. AST-120 use was reported to decrease the IS levels in a dose-dependent manner in nondialysis CKD patients^8^. This report suggests that AST-120 use may have a greater beneficial effect on the decrease in PBUTs in anuric patients undergoing maintenance dialysis treatment because they have higher levels of PBUTs^1^,^5^.

In this study, we investigated whether oral administration of AST-120 induced the reduction of several PBUTs in anuric patients undergoing maintenance haemodialysis. We also assessed the subsequent effects of AST-120 use on some oxidative stress markers.

## Results

### Baseline characteristics

The median age of the patients was 63.0 (58.3–65.0) years. Women accounted for 40.0% of the study population. The median duration of dialysis was 15.5 (8.0–20.8) years, and the patients had no residual kidney function. The patients were divided into 2 groups, i.e. Group I (n = 10) and Group II (n = 10), which were similar in terms of demographic, dialysis, and laboratory characteristics (Table 1).

### Uraemic toxins in haemodialysis patients treated with AST-120

**Figure 1:**
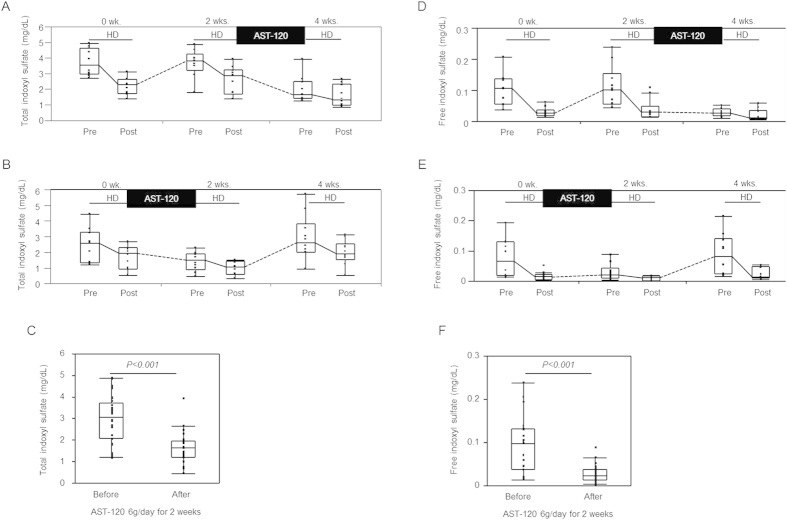
Serum levels of total and free indoxyl sulfate (IS) in maintenance haemodialysis patients receiving AST-120 treatment. Serial changes in the serum levels of total IS ((**A**) Group I, (**B**) Group II, n = 10 per group) and free IS ((**D**) Group I, (**E**) Group II, n = 10 per group), and a comparison between the serum levels before and those after AST-120 use in the predialysis session ((**C**) total IS, (**F**) free IS, n = 20). P values were calculated using the Wilcoxon signed-rank test.

During the 4-week study, haemodialysis patients received AST-120 (6 g/day) in the last 2 weeks (Group I) or the first 2 weeks (Group II) of the study. No adverse effects of AST-120, such as constipation, loss of appetite, and nausea, were reported in the participants by using a questionnaire. In Group I, the serum levels of total and free IS before the dialysis session were 3.09 mg/dL (2.80–4.62 mg/dL) and 0.106 mg/dL (0.055–0.136 mg/dL), respectively, and the protein-binding rate was 97.1% (96.6–97.9%), which is similar to that reported by a previous study^5^. A haemodialysis session decreased the total and free IS levels to 2.16 mg/dL (1.59–2.61 mg/dL) and 0.026 mg/dL (0.018–0.036 mg/dL), respectively, whereas regular haemodialysis treatment maintained a high serum level of IS in the predialysis session. However, use of AST-120 (6 g/day) for 2 weeks dramatically decreased the total and free IS levels in the predialysis session (total IS: 1.62 mg/dL [1.31–2.48 mg/dL]; free IS: 0.027 mg/dL [0.017–0.040 mg/dL]), and the low levels were maintained between dialysis sessions (Fig. 1A,D). In Group II, use of AST-120 (6 g/day) for 2 weeks decreased the total and free IS levels in the predialysis session (total IS: 2.55 mg/dL [1.34–3.27 mg/dL] before treatment and 1.49 mg/dL [0.90–1.91 mg/dL] after treatment; free IS: 0.067 mg/dL [0.018–0.130 mg/dL] before treatment and 0.020 mg/dL [0.009–0.043 mg/dL] after treatment). However, the total and free IS levels increased after the discontinuation of AST-120 (Fig. 1B,E). The total and free IS levels in Groups I and II in the predialysis session decreased significantly with AST-120 treatment (total IS: 1.63 mg/dL [1.19–1.98 mg/dL] vs. 3.06 mg/dL [2.08–3.74 mg/dL] before treatment, P < 0.001; free IS: 0.023 mg/dL [0.013–0.038 mg/dL] vs. 0.098 mg/dL [0.038–0.131 mg/dL] before treatment, P < 0.001), and the reduction rates of total and free IS were 45.7% (33.2–50.5%) and 70.4% (44.8–79.8%), respectively (Fig. 1C,F). When the association between the initial serum levels of IS and the reduction ratio with AST-120 treatment was evaluated, free IS, but not total IS, showed a statistically significant correlation with the reduction ratio (free IS: Rho = 0.713, P < 0.001; total IS: Rho = −0.011, P = 0.965).

**Figure 2:**
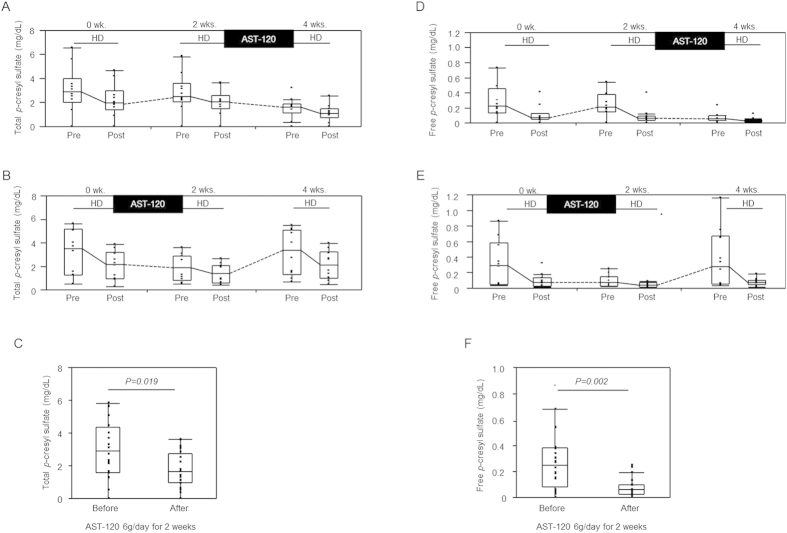
Serum levels of total and free *p-*cresyl sulfate (PCS) in maintenance haemodialysis patients receiving AST-120 treatment. Serial changes in the serum levels of total PCS ((**A**) Group I, (**B**) Group II, n = 10 per group) and free PCS ((**D**) Group I, (**E**) Group II, n = 10 per group), and a comparison between the serum levels before and those after AST-120 use in the predialysis session ((**C**) total IS, (**F**) free IS, n = 20). P values were calculated using the Wilcoxon signed-rank test.

The effects of AST-120 on the serum levels of PCS showed the same trend as those of AST-120 on the serum levels of IS in Group I (Fig. 2A,D) and Group II (Fig. 2B,E). The serum levels of PCS also decreased with AST-120 use (total PCS: 1.66 mg/dL [0.96–2.77 mg/dL] vs. 2.90 mg/dL [1.59–4.34 mg/dL] before treatment, P = 0.019; free PCS: 0.061 mg/dL [0.028–0.100 mg/dL] vs. 0.250 mg/dL [0.082–0.384 mg/dL] before treatment, P = 0.002) (Fig. 2C,F), and the reduction rates of total and free PCS with AST-120 treatment were 31.1% (25.0–48.0%) and 63.5% (49.3–70.9%), respectively. When the association between the initial serum levels of PCS and the reduction ratio with AST-120 treatment was evaluated, free PCS, but not total PCS, showed a statistically significant correlation with the reduction ratio (free PCS: Rho = 0.584, P = 0.007; total PCS: Rho = 0.438, P = 0.054).

**Figure 3:**
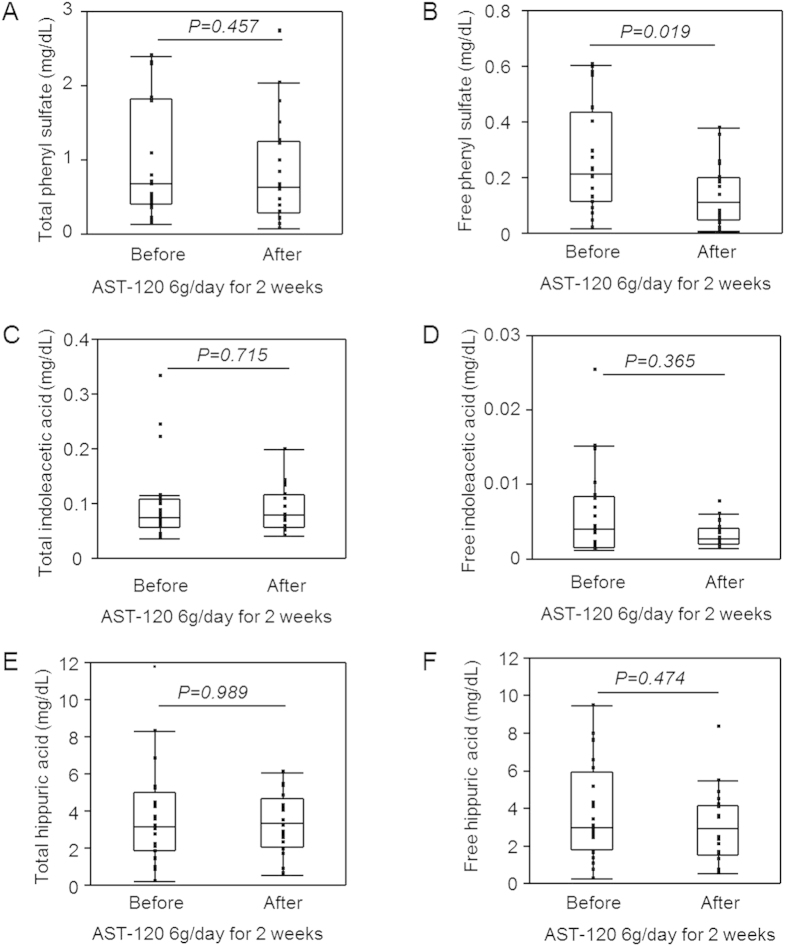
Serum levels of total and free phenyl sulfate (PS), indoleacetic acid (IAA), and hippuric acid (HA) in maintenance haemodialysis patients receiving AST-120 treatment. A comparison between the total and free PS, IAA, and HA serum levels before and those after AST-120 use in the predialysis session ((**A**) total PS, (**B**) free PS, (**C**) total IAA, (**D**) free IAA, (**E**) total HA, (**F**) free HA, n = 20). P values were calculated using the Wilcoxon signed-rank test.

Free phenyl sulfate (PS) levels decreased significantly with AST-120 treatment (0.11 mg/dL [0.05–0.20 mg/dL] vs. 0.22 mg/dL [0.11–0.44 mg/dL] before treatment, P = 0.019), whereas total PS levels did not change (0.63 mg/dL [0.29–1.26 mg/dL] vs. 0.69 mg/dL [0.40–1.82 mg/dL] before treatment, P = 0.456) (Fig. 3A,B, Supplementary Fig. 1). On the other hand, both total and free indoleacetic acid (IAA) and hippuric acid (HA) levels did not change with AST-120 treatment (Fig. 3C–F, Supplementary Figs 2 and 3).

To understand the effects of AST-120 on the changes in low- and intermediate-molecular-weight water-soluble molecules, urea nitrogen and β_2_-microglobulin levels were measured during the study, and AST-120 treatment did not decrease the levels of both urea nitrogen (62.5 mg/dL [55.2–70.8 mg/dL] vs. 65.2 mg/dL [57.4–72.4 mg/dL] before treatment, P = 0.525) and β_2_-microglobulin (24.2 mg/L [21.0–26.8 mg/L] vs. 24.5 mg/dL [21.4–26.8 mg/L] before treatment, P = 0.978] (Supplemental Fig. 4).

These results indicate that the use of AST-120 induced a dramatic reduction of some PBUTs, such as IS (total and free), PCS (total and free), and PS (free) in patients on maintenance haemodialysis treatment.

### Oxidative stress in patients undergoing haemodialysis treated with AST-120

**Figure 4:**
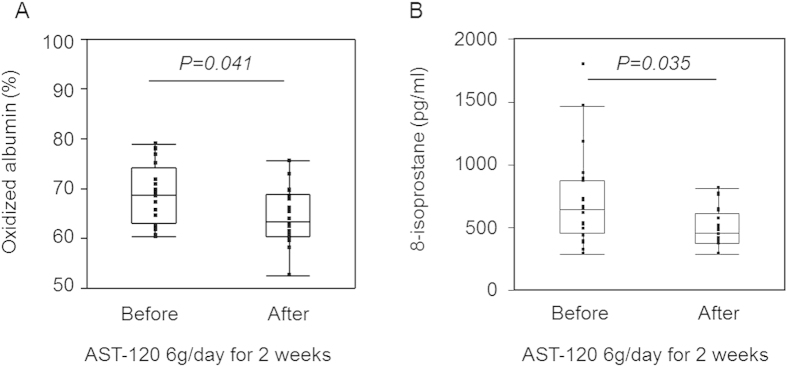
Changes in the levels of oxidative stress markers in maintenance haemodialysis patients receiving AST-120 treatment. A comparison between the oxidized albumin (**A**) and 8-isoprostane (**B**) levels before and those after AST-120 use in the predialysis session. P values were calculated using the Wilcoxon signed-rank test.

We also evaluated the changes in the oxidative stress markers with AST-120 treatment in patients undergoing maintenance haemodialysis. Use of AST-120 induced significant reduction in oxidized albumin content [63.4% (60.3–69.0%) vs. 68.5% (63.0–74.3%) before AST-120 treatment, P = 0.041, Fig. 4A) as well as 8-isoprostane [458.1 pg/mL (380.7–615.6 pg/mL) vs. 642.4 pg/mL (450.7–880.6 pg/mL) before AST-120 treatment, P = 0.035, Fig. 4B]. These results indicate that the use of AST-120 was related to improvement of oxidative stress in patients undergoing maintenance haemodialysis treatment.

## Discussion

In this study, anuric patients undergoing maintenance haemodialysis treatment were orally administered the charcoal adsorbent AST-120 which induced a dramatic the levels of some PBUTs. Furthermore, AST-120 use was associated with the improvement in some oxidative stress markers.

PBUTs are accumulated in CKD patients, especially in those undergoing dialysis treatment^1^,^5^. High serum levels of PBUTs, such as IS, PCS, and IAA, are associated with higher rates of cardiovascular and all-cause mortality in dialysis patients^1^,^2^,^9^. The mechanisms through which PBUTs induce various CKD-related complications including cardiovascular disease are still incompletely understood; basic research suggests a direct interaction between those uraemic toxins and cardiovascular tissues^10^,^11^ with aggravation of oxidative stress^3^,^6^,^7^. However, conventional haemodialysis is limited in its ability to remove PBUTs, such as IS, IAA, and HA, even with convective therapies including high-flux haemodialysis, haemofiltration, and haemodiafiltration^4^,^12^, although recent preliminary data suggested that change in pH during haemodialysis may increase the removal of IS (Chun J, *et al.* *Kidney Week 2014*, 861A [2014]). Thus, an additional blood purification strategy is needed to decrease PBUT levels, which will lead to better survival in dialysis patients.

The oral charcoal adsorbent AST-120 prevents IS synthesis by inhibiting the gastrointestinal uptake of indole in nondialysis CKD patients^8^. Although the randomised placebo-controlled EPPIC-1 and EPPIC-2 trials did not show the beneficial effects of the use of AST-120 (9 g/day) on the endpoints including dialysis initiation, kidney transplantation, and serum creatinine doubling in patients with stage 4 CKD^13^, we believe that these effects will be much more obvious in maintenance dialysis patients with a high level of accumulated PBUTs. Thus, we examined whether oral administration of AST-120 had an additive effect of PBUT reduction in anuric patients undergoing maintenance haemodialysis. As a result, AST-120 use with conventional haemodialysis induced a dramatic decrease in the IS (total and free), PCS (total and free), and PS (free) levels (Figs 1, 2, 3, Supplementary Fig. 1). These beneficial effects may improve survival in maintenance haemodialysis patients because high serum levels of total IS are reported to be associated with higher mortality in CKD patients^1^. The decrease in the free IS and PCS levels was more obvious than that in the total IS and PCS levels because AST-120 inhibits the absorption of indole or *p-*cresol, derived from dietary tryptophan and tyrosine respectively across the intestinal tract^14^,^15^,^16^, which leads to the inhibition of free IS and PCS production. Another possible mechanism is that the remaining IS and PCS contents after AST-120 treatment tend to bind to proteins, leading to an increase in the protein-binding rate. The binding saturation of free vs. total solute concentrations and competitive binding of different solutes at the same protein-binding sites should be discussed in future studies. The levels of free PS, but not total PS, decreased with AST-120 treatment (Fig. 3A,B, Supplementary Fig. 1). Similar to PCS^14^, PS is produced from tyrosine in the intestinal tract, and the effects of AST-120 use may be partial for total PS because compared with the amounts of IS (reduction rate 34.3% [27.0–42.8%], Fig. 1A,B) and PCS (reduction rate: 32.3% [21.9–36.5%], Fig. 2A,B), large amounts of PS were removed using haemodialysis (reduction rate: 71.3% [63.5–75.7%], Supplementary Fig. 1a,b). On the other hand, AST-120 use did not change the serum levels of IAA and HA (Fig. 3C–F, Supplementary Figs 2 and 3). The mechanism of gastrointestinal production and uptake of those molecules may not be as crucial for those molecules. A recent study showed that an increase in the levels of dietary fibre led to a decrease in the plasma levels of IS (total and free) and PCS (free) in haemodialysis patients^17^. A previous study showed that colectomy decreased the plasma levels of IS and PCS, but not those of HA, in patients undergoing dialysis treatment^18^. Thus, oral administration of AST-120 showed effects mainly on uraemic toxins of colonic origin, such as IS, PCS, and PS. Lee *et al.* also reported the effects of AST-120 on the reduction of IS and PCS levels in patients undergoing peritoneal dialysis or haemodialysis^19^. They analysed the data obtained from haemodialysis and peritoneal dialysis patients with residual kidney function; however, the additional effects of AST-120 on the decrease in PBUT levels in dialysis patients are unclear. In contrast, we examined the effects of AST-120 on various types of PBUTs in maintenance haemodialysis patients with no residual kidney function both before and after the dialysis session. Moreover, we observed that the PBUT levels increased again after discontinuation of the treatment. Thus, we found that AST-120 use with conventional dialysis treatment in haemodialysis patients induced a strong and continuous reduction in the levels of PBUTs of colonic origin.

AST-120 was also associated with the improvement of some oxidative stress markers (Fig. 4). This was probably because of the subsequent effects after the decrease in the PBUT levels. In an animal model, AST-120 administration modulated kidney injury-induced cardiac fibrosis through oxidative stress^20^. In another animal model, AST-120 administration inhibited kidney injury-induced acceleration of atherosclerosis with a decrease in IS deposition as well as inflammatory cytokine expressions in lesions^21^. A clinical study reported that AST-120 use improved flow-mediated endothelium-dependent vasodilatation and the oxidized glutathione/glutathione ratio in nondialysis CKD patients^22^. Taken together, a decrease in the PBUT levels with the use of AST-120 and conventional haemodialysis may lead to improvement of oxidative stress in maintenance haemodialysis patients.

This study has some limitations. This was a crossover trial, not a randomised placebo-controlled study. The sample size was small and the treatment duration was short, which limited our understanding of the subsequent effects of AST-120, such as oxidative stress and inflammatory reactions. Moreover, serum samples were collected only at 0, 2, and 4 weeks during the study, which limited our understanding of the time taken for the serum concentrations to be affected. Long-term observation of maintenance haemodialysis patients will be needed to understand the effects of AST-120 on uraemia-related complications including cardiovascular outcomes. However, despite the small sample size and short treatment duration, our data suggest that oral charcoal adsorbents may be used as an additional blood purification therapy to reduce PBUTs in maintenance dialysis patients.

In conclusion, oral administration of AST-120 was effective in the continuous reduction of some PBUTs and was associated with the improvement of oxidative stress in maintenance haemodialysis treatment. This adsorption treatment may be an additional blood purification therapy to prevent uraemia-related complications.

## Methods

### Study design and patients

We recruited 20 patients undergoing maintenance haemodialysis treatment who had no residual kidney function, from a single dialysis unit. All eligible patients received 4–5 h of haemodialysis thrice weekly with standard bicarbonate dialysate (Na^+^: 140 mEq/L, K^+^: 2.0 mEq/L, Ca^2+^: 2.75 mEq/L, Mg^2+^: 1.0 mEq/L, Cl^−^: 112.25 mEq/L, HCO_3_- 27.5 mEq/L) and 1.6–2.1 m^2^ dialyzers with synthetic membranes, polysulfone or polyethersulfone. The blood flow was 180–250 ml/min, and dialysate flow was 500 mL/min. Dialysis adequacy was estimated by Kt/V_urea_. Patients who had acute inflammatory disease, underwent haemodialysis for <1 year, and/or were <20 years old were excluded. To observe differences in patient characteristics before and after the intervention, the patients were divided into 2 groups, i.e. Group I and Group II, which did not differ in terms of age, sex, and dialysis duration. In Group I patients, AST-120 was not administered for the first 2 weeks and then was administered at 6 g/day for the last 2 weeks. In Group II patients, AST-120 was administered for the first 2 weeks and then was not administered for the last 2 weeks. In this study, AST-120 was administered at 6 g/day in haemodialysis patients because in Japan, the regular dose of AST-120 in predialysis CKD patients is 6 g/day^23^. Serum samples were obtained from patients before and after the dialysis sessions at 0, 2, and 4 weeks. Baseline data such as age, sex, body mass index, cause of end-stage kidney disease, systolic blood pressure, duration of dialysis treatment, single pool Kt/V_urea_, and serum levels of urea nitrogen, creatinine, albumin, blood haemoglobin, calcium, phosphorus, intact parathyroid hormone, and C-reactive protein at the predialysis session were also measured. This study adhered to the Declaration of Helsinki and was approved by the Central Ethics Committee at Niigata University. It was registered at the University Hospital Medical Information Network Center (UMIN000009618). All participants provided written informed consent.

### Measurement of uraemic toxins

Serum samples were frozen at −30 °C immediately after collection from patients and thawed just before the measurement of uraemic toxins, including low- and intermediate-molecular-weight water-soluble molecules and protein-bound solutions. In the case of PBUTs, levels of both the total and free forms of 5 endogenous metabolites such as IS, PCS, PS, IAA, and HA were measured by mass spectrometry as described previously^5^. Urea nitrogen and β_2_-microglobulin levels were also measured during the study.

### Measurement of oxidative stress

Chromatographic analysis of serum albumin in haemodialysis patients was performed as described previously^24^. High-performance liquid chromatography (HPLC) analysis of 5 μL aliquots of each serum sample was performed using a Shodex Asahipak ES-502N column (Showa Denko Co., Ltd., Tokyo, Japan; column temperature, 35 ± 0.5 °C). The HPLC system was composed of an intelligent pump (L-6200) equipped with a gradient programmer and an F-1050 fluorescence detector (Hitachi Co., Ltd., Tokyo, Japan). Elution was performed using a linear gradient with increasing ethanol concentrations from 0% to 5% for serum in 0.05 mol/L of sodium acetate and 0.40 mol/L of sodium sulfate mixture (pH 4.85) at a flow rate of 1.0 mL/min. From the HPLC profiles of serum albumin, the value of oxidized albumin content was estimated by dividing the area of oxidized albumin by the total area corresponding to albumin. The serum level of 8-isoprostane was measured using an enzyme-linked immunosorbent assay kit (Detroit R&D Inc. Detroit, MI, USA).

### Statistical analyses

The sample size was calculated using published results showing 2.11 ± 0.93 mg/dL as the mean value and standard deviation for CKD patients with a high risk of mortality^1^. To detect an 80% difference, we estimated 15 participants to achieve 90% statistical power with a 2-sided significance level of 5%. Regarding patient characteristics, continuous variables were expressed as medians (interquartile range: IQR). Regarding the uraemic toxin levels, data were expressed as medians (IQR), and the Wilcoxon signed-rank test was used for comparisons between the levels before and those after AST-120 use. The Spearman rank correlation test was used to assess the correlation between the initial serum levels of PBUTs and the reduction ratio. All statistical analyses were performed using JMP7 (SAS Institute; Cary; NC, USA). A two-sided significance level of 5% was considered statistically significant.

## Additional Information

**How to cite this article**: Yamamoto, S. *et al.* Continuous Reduction of Protein-Bound Uraemic Toxins with Improved Oxidative Stress by Using the Oral Charcoal Adsorbent AST-120 in Haemodialysis Patients. *Sci. Rep.* **5**, 14381; doi: 10.1038/srep14381 (2015).

## Supplementary Material

Supplementary Information

## Figures and Tables

**Table 1:** Clinical characteristics of the participants.

	Groups I and II	Group I	Group II
*Demographic and clinical characteristics*			
Number of patients	20	10	10
Age, years	63.0 (58.3–65.0)	63.0 (58.3–65.3)	62.5 (57.8–65.8)
Sex, male/female	12/8	6/4	6/4
Body mass index, kg/m^2^	21.3 (19.8–22.4)	21.6 (19.8–23.3)	21.1 (19.2–22.4)
Cause of ESKD, CGN/DM/others	14/2/4	8/0/2	6/2/2
Systolic blood pressure, mmHg	140 (132–147)	138 (132–143)	148 (130–150)
*Dialysis*			
Duration of dialysis, years	15.5 (8.0–20.8)	15.0 (7.8–20.2)	16.0 (8.0–22.3)
Kt/V_urea_	1.71 (1.44–1.90)	1.55 (1.29–1.91)	1.76 (1.63–1.92)
*Laboratory data*			
Urea nitrogen, mg/dL	62.5 (56.5–77.5)	67.0 (54.3–78.3)	61.5 (58.3–76.8)
Creatinine, mg/dL	12.5 (10.2–13.8)	12.5 (11.3–14.0)	12.2 (9.4–13.7)
Albumin, g/dL	4.1 (3.8–4.3)	4.2 (4.0–4.3)	4.0 (3.8–4.4)
Blood haemoglobin, g/dL	11.6 (10.8–12.2)	11.3 (10.5–12.0)	11.8 (11.0–12.4)
Calcium, mg/dL	9.2 (8.6–9.5)	9.2 (8.6–9.6)	9.2 (8.7–9.4)
Phosphorus, mg/dL	5.2 (4.7–6.3)	5.3 (5.0–6.3)	4.8 (4.7–6.4)
Intact PTH, pg/mL	145.5 (84.5–225.3)	147.5 (109.3–259.3)	141.5 (34.3–189.8)
C-reactive protein, mg/dL	0.06 (0.03–0.14)	0.06 (0.03–0.16)	0.07 (0.04–0.14)

CGN, chronic glomerulonephritis; DM, diabetes mellitus; ESKD, end-stage kidney disease; PTH, parathyroid hormone.
