# Supplementary material for: Continuous Reduction of Protein-Bound Uraemic Toxins with Improved Oxidative Stress by Using the Oral Charcoal Adsorbent AST-120 in Haemodialysis Patients
Source: Sci Rep. 2015 Sep 23;5:14381. doi: 10.1038/srep14381 (PMC4585768; doi:10.1038/srep14381)
Supplement: Supplementary Information [file srep14381-s1.pdf]

Supplementary Information

Continuous Reduction of Protein-Bound Uraemic Toxins with Improved Oxidative Stress by Using the Oral Charcoal Adsorbent AST-120 in Haemodialysis Patients

Suguru Yamamoto[1*], Junichiro J. Kazama[1,2], Kentaro Omori[3], Koji Matsuo[1], Yoshimitsu Takahashi[1], Kazuko Kawamura[1], Takayuki Matsuto[4], Hiroshi Watanabe[5], Toru Maruyama[5], and Ichiei Narita[1]

[1]Division of Clinical Nephrology and Rheumatology and [4]Division of Clinical Preventive Medicine, Niigata University Graduate School of Medical and Dental Sciences, Niigata 951-8510, Japan

[2]Division of Blood Purification Therapy, Niigata University Medical and Dental Hospital, Niigata 951-8520, Japan

[3]Omori Clinic, Niigata 950-0909, Japan

[5]Department of Biopharmaceutics, Graduate School of Pharmaceutical Sciences, Kumamoto University, Kumamoto 862-0973, Japan

*Correspondence to: Suguru Yamamoto, Division of Clinical Nephrology and Rheumatology, Niigata University Graduate School of Medical and Dental Science, 1-757 Asahimachi-dori, Chuo-ku, Niigata 951-8510, Japan

Tel: +81-25-227-2200, Fax: +81-25-227-0775

E-mail: yamamots@med.niigata-u.ac.jp

Supplemental Figure 1. Serum levels of total and free phenyl sulfate (PS) with AST-120 treatment in patients undergoing maintenance haemodialysis (HD) treatment. Serial changes in the serum levels of total PS (a: Group I, b: Group II, n = 10 each) and free PS (c: Group I, d: Group II, n = 10 each) with AST-120 treatment.

Supplemental Figure 2. Serum levels of total and free indoleacetic acid (IAA) with AST-120 treatment in patients undergoing maintenance haemodialysis (HD) treatment. Serial changes in the serum levels of total IAA (a: Group I, b: Group II, n = 10 each) and free IAA (c: Group I, d: Group II, n = 10 each) with AST-120 treatment.

Supplemental Figure 3. Serum levels of total and free hippuric acid (HA) with AST-120 treatment in patients undergoing maintenance haemodialysis (HD) treatment. Serial changes in the serum levels of total HA (a: Group I, b: Group II, n = 10 each) and free HA (c: Group I, d: Group II, n = 10 each) with AST-120 treatment.

Supplemental Figure 4. Serum levels of urea nitrogen and β_2-microglobulin with AST-120 treatment in patients undergoing maintenance haemodialysis (HD) treatment. Serial changes in the serum levels of urea nitrogen (a: Group I and b: Group II, n = 10 each), and β_2-microglobulin (d: Group I and e: Group II, n = 10 each), and the comparison between before and after use of AST-120 at the pre-dialysis session (c: urea nitrogen, f: β_2-microglobulin, n = 20). P values from the Wilcoxon rank sum test.

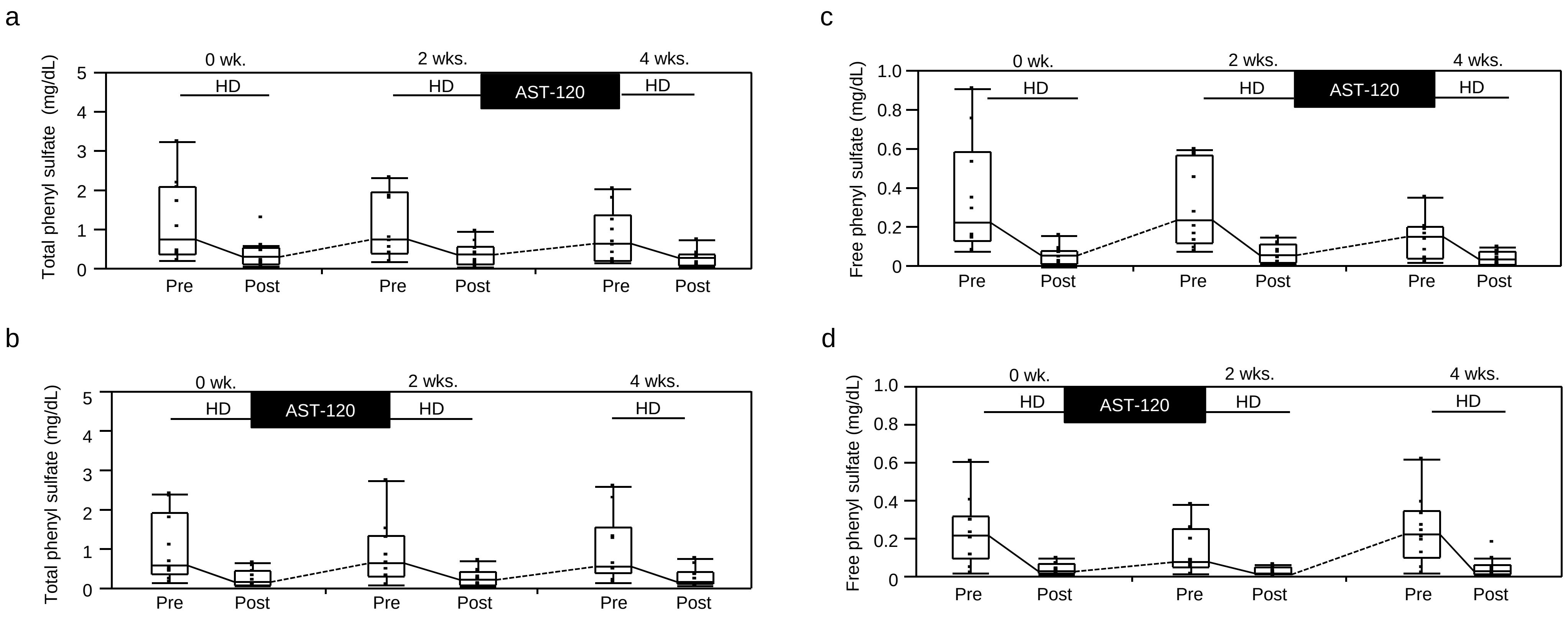

Yamamoto S et al. Supplemental Figure 1

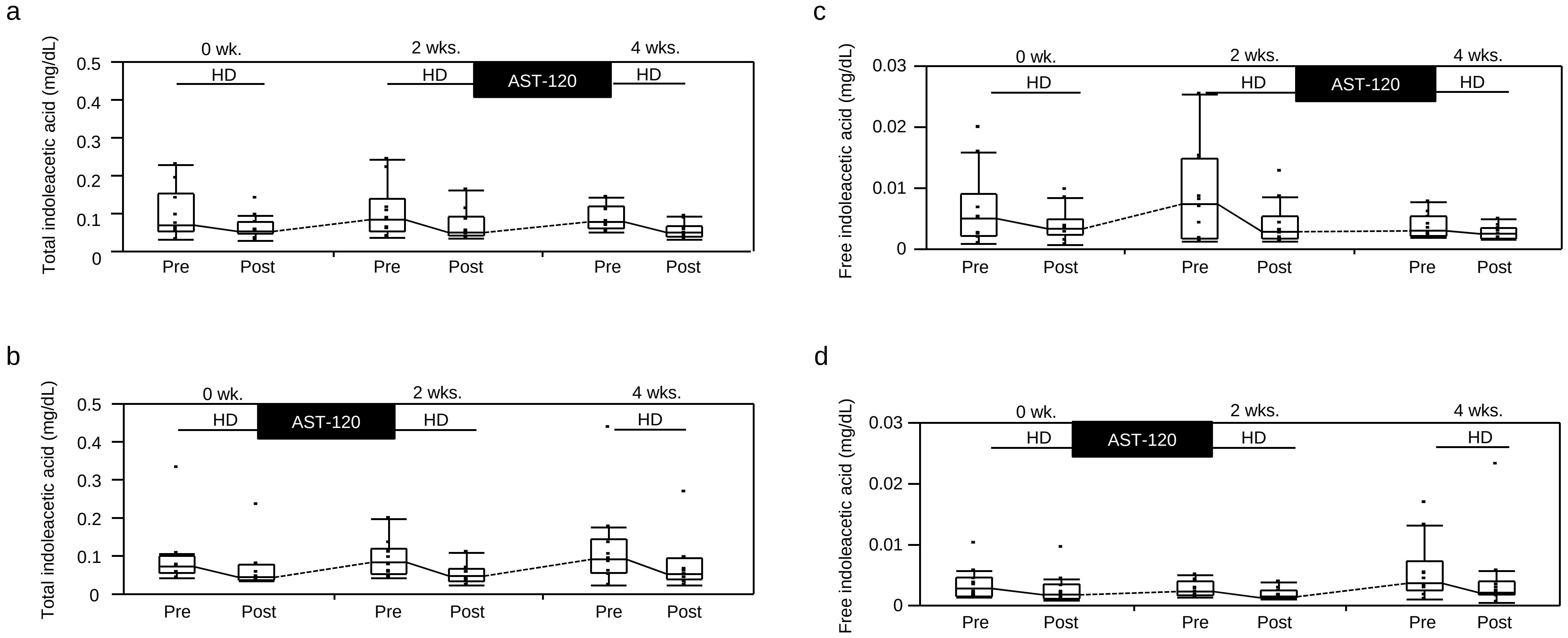

Yamamoto S et al. Supplemental Figure 2

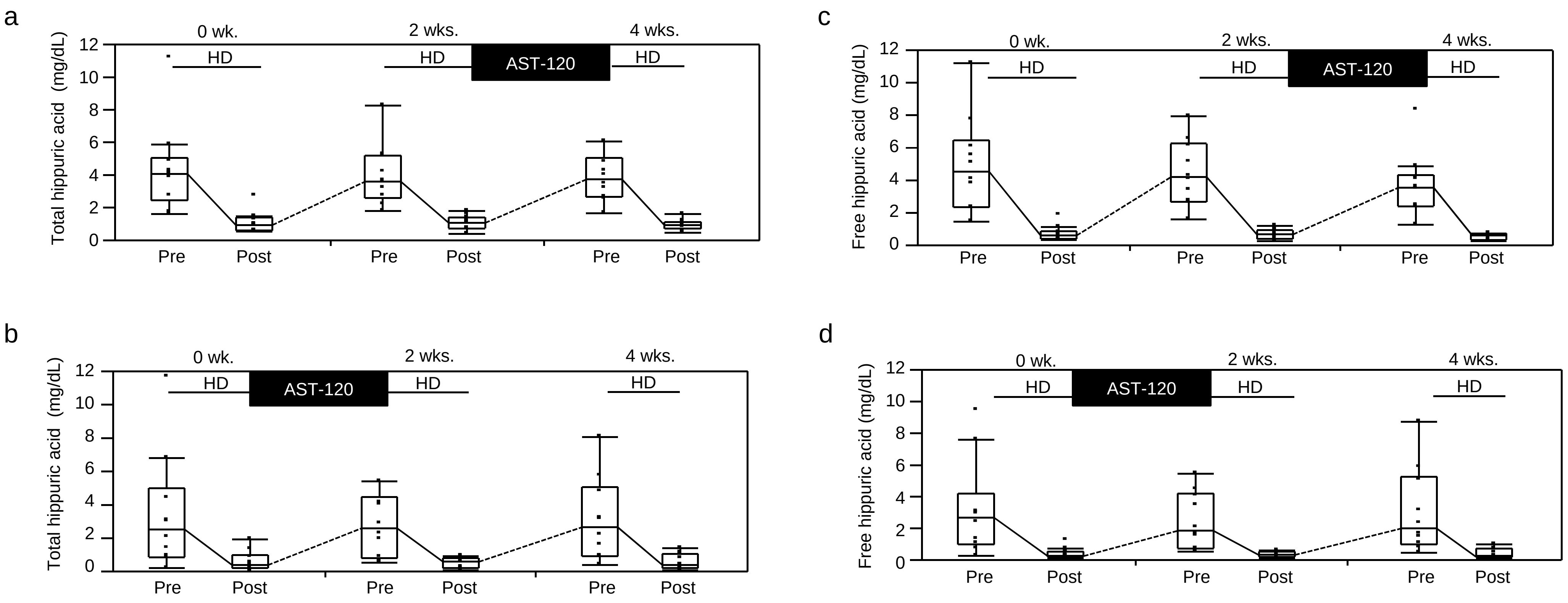

Yamamoto S et al. Supplemental Figure 3

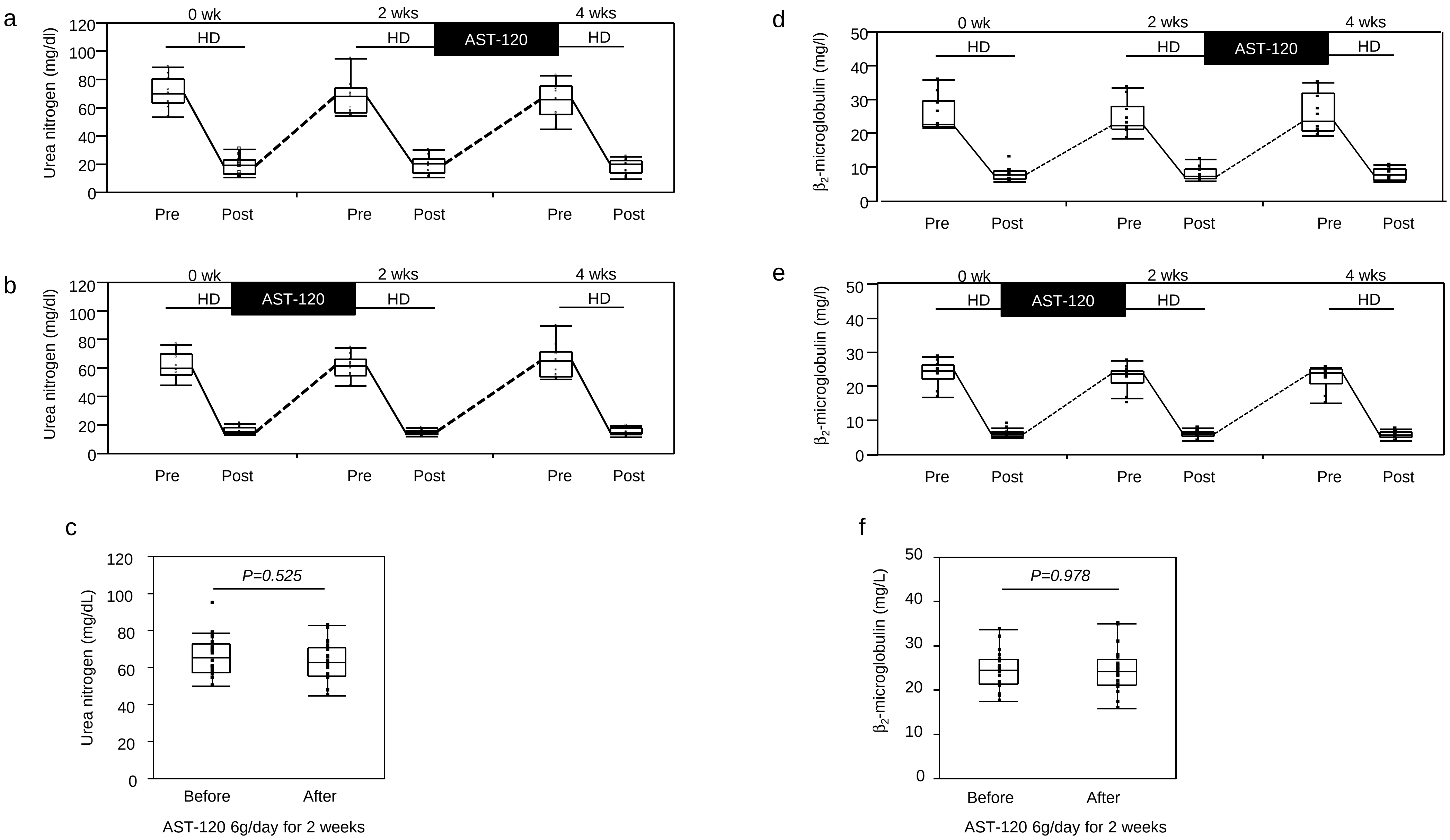

Yamamoto S et al. Supplemental Figure 4
